# Responsibility endowing power: A theoretical framework of health empowerment for Chinese older people with chronic illness

**DOI:** 10.1002/nop2.2236

**Published:** 2024-07-03

**Authors:** Heng Zhang, Hao Zhang, Jie Peng

**Affiliations:** ^1^ Nanjing University of Chinese Medicine Nanjing China; ^2^ BengBu Central Hospital BengBu China

**Keywords:** chronic illness, grounded theory, health empowerment, nursing, older people, theory framework

## Abstract

**Aim:**

To construct a health empowerment framework for the Chinese older people with chronic conditions.

**Design:**

A Strussian grounded theory design was selected to generate the theoretical framework.

**Methods:**

Data were collected from 53 community‐dwelling older people with chronic conditions in China between November 2017 and August 2019, via semi‐structured interviews and with participating observation. The constant comparative method identified the key categories.

**Results:**

‘Responsibility endowing power’, the health empowerment core theme, was defined as initiating, performing and realizing responsibility towards health through the interaction between the self, family and society. The framework enriches the meaning of health empowerment, changing older people's nursing practice.

## 1 INTRODUCTION

The aging of the Chinese population with a high prevalence of chronic conditions is having an impact on society at large (Xia & Li, 2018). For individuals with long‐term health problems, well‐being is not simply the treatment of symptoms, but living a more meaningful life (Thorne, 1999). Funnell and Anderson (2004) asserted that ‘knowing about an illness is not the same as knowing about a person's life’, highlighting the nature of patient empowerment, consistent with the inner strength of individuals in self‐management or a meaningful life rather than simply changing behaviour (Leyshon, 2002; Lundman et al., 2010; Thorne et al., 2000). Increasingly, health empowerment attaches importance to the dialogue between health professionals and clients characterized by mutual respect and positive participation in the decision‐making to strengthen self‐awareness and self‐esteem (Anderson & Funnell, 2010). Crawford Shearer and Reed (2004), Shearer (2009), Shearer et al. (2009) demonstrated that successful patient empowerment should root in a mutual participatory process involving the synthesis of individual capacity and external social‐contextual resources. According to Tucker et al. (2018), individuals who possess the power to modify their social or environmental factors can effectively promote healthy behaviour. This includes being able to make informed treatment decisions and having trust in their health care providers (Schulz & Nakamoto, 2013).

Some studies that focus on patient empowerment address more specifically individual inherent capacity to reconstruct a positive life in spite of harsh conditions. Patient empowerment signifies confidence, higher self‐efficacy or health locus of control in self‐management among people with chronic illness (Náfrádi et al., 2017; Wang et al., 2022). Aujoulat et al. (2008) described patient empowerment as a dual process of gaining or relinquishing control to integrate illness as part of a reconciled self. Some nursing theories, derived from the experience of persons living with chronic conditions, such as Health‐within‐illness (Moch, 1998), Inner Strength (Smith et al., 2019) and Transition (Meleis, 2012), reflect a positive health philosophy to live in harmony with health problems and reformulate a new self to achieve empowerment. Empowered patients with high health literacy might believe that they can make intelligent non‐adherence to elect freely to interrupt or forgo a therapy, suggesting a controversial and intertwined relationship among health literacy adherence, and patient empowerment (Prigge et al., 2015).

In conclusion, empowerment is more like a philosophy that encompasses diversity, complexity and controversy (Pekonen et al., 2020; Shearer et al., 2012). However, it is indisputable that patient empowerment is both a process and an outcome related to awareness, capacity, autonomy and relationship. There are limited studies on health empowerment in Chinese chronically ill older population. How do Chinese older adults regard their living experience with chronic conditions, their capacities, decision‐making in disease management and the relationship with health care staff? A matter of concern is whether the older population is empowered to effectively manage the chronic conditions to attain well‐being, and what could facilitate or impede empowerment.

## 2 AIMS

The study aimed to explore the living experience of Chinese older adults with chronic conditions and to develop a health empowerment theoretical framework grounded in China's context, allowing for further empowerment intervention.

## 3 METHOD

### 3.1 Qualitative design and research paradigm

A Straussian grounded theory using a semi‐structured interview with participating observation was selected to generate the theoretical framework (Corbin & Strauss, 2008). As we know, there are three common approaches for grounded theory studies including classic, Straussian and constructivist. Although the three types are similar, the classic grounded theory should neither conduct a preliminary literature review nor begin with a pre‐determined research question (Vander Linden & Palmieri, 2021). The Straussian grounded theory asserts that researchers should review appropriate literatures before going into the field and encourage particular topic area in research question. Unlike the other two types, however, the Straussian grounded theory integrates the capacity for reflection, comparison and creativity to improve theoretical sensitivity without ignoring the objectivity or independence of the researcher (Kim et al., 2005; Rieger, 2019). In our study, the research issues derived from both specific professional practice in population aging and extant literature informing health empowerment, ensuring the researcher was not a blank slate and had an interpretive role in later analysis (Corbin & Strauss, 2015), which was congruent with the Straussian grounded theory approach.

### 3.2 Participants and sampling

Older people with chronic conditions were recruited by purposive sampling and subsequent theory sampling from Shanghai, Nanjing and Bengbu in Eastern China. The inclusion criteria were age over 60 years, a minimum of one self‐reported chronic condition, being physically able and willing to participate and being able to give informed consent. Individuals with dementia and a lack of capacity for communication were excluded.

First, a convenience sample from an adult day care centre in Shanghai, a community healthcare centre in Nanjing and a clinic of a tertiary hospital in Bengbu was selected by contacting healthcare professionals. Some of the participants were institutionalized, and some were outpatients and lived at home generally. Participants from the day care centre went home at 4 pm and returned to the centre at 8 am daily. A heterogenic sampling of participants is recommended to maximize the variations of experiences and saturate findings in the group studied (Mohammadi et al., 2020). Participants were enrolled subsequently according to different diseases or progress, various educational backgrounds, religious beliefs and economic status, so as to explore the correlation between these factors for theories derivation. As the elements of the empowerment framework gradually emerged, theoretical sampling was driven by the emerging categories and hypotheses for theoretical elaboration in the empirical data (Jeon, 2004). Actually, sampling from the first‐ (Shanghai), second‐ (Nanjing) and third‐tier (Bengbu) cities based on the influence of urban economic development level on the life of older people was a type of theoretical sampling. Another example, some participants wished their offspring be medical personnel so that they could readily access health‐related help. This highlighted the issue of accessibility of health resources. Eight participants living at home whose children were medical staff were subsequently enrolled.

### 3.3 Ethical considerations

The research protocol was approved by the University Ethics Committee of Nanjing University of Chinese Medicine in order to protect the participants. All participants signed a consent form before data collection. They were fully informed of the purpose of the study and the length of the interview. The recordings and transcripts of all participants were assigned anonymous alphabetical codes for analysis purposes only, as promised.

### 3.4 Data collection

Data were collected through in‐depth interviews and participating observation between November 2017 and August 2019. The first author with expertise in clinical practice and prior training in qualitative methods conducted all interviews, and observed the older adults' daily behaviour to capture non‐verbal information such as the expressions, tone, sighs and other body language and behaviours of the interviewees, and recorded them in the field notes, so as to verify the consistency of acquired information. Also, the first author volunteered to be a member of the staff  in a day care centre in Shanghai, immersed in the daily caring for older people and observed the behaviours and attitudes of older people to confirm the mentioned in the interview. Open‐ended questions were designed to identify concepts related to empowerment. Considering the abstractness of the word ‘empowerment’, the first author invited the participants to describe their experience of living with the disease and self‐management strategies and identified the related themes. After a two‐case pilot interview, the researcher revised the interview guideline. During the formal interview, the interview methods and order were adjusted according to the differences in the interviewees' understanding level and the emphasis on data analysis (Table 1).

**TABLE 1:** Examples of questions and probes included in the semi‐structured interview guide.

What was your experience when you were diagnosed with the disease?
Probes: What kind of change occurred in your mind or behaviour?
What does it mean for you to be healthy?
What would you describe as your greatest concern regarding your health?
2 Could you control your illness? How did you perceive the sense of control? How did you improve control over your illness and maintain health?
Probes: What strategies do you use to manage health or improve your coping mechanisms?
Who or what has been most/least helpful during the process of management of your health?
What factors are limiting your health self‐management?
3 What type of information or pattern did you prefer if you were invited to participate in a health management program?

Interviews were conducted in the quiet rest room of the day care centre or the living room of the participants' homes. Each interview lasted between 40 min and 1.5 h, was tape‐recorded with the participants' permission and transcribed verbatim in Chinese shortly afterwards. Every record was transcribed verbatim within 24 h, noting the participants' current pauses, voice expressions and body language. One author with practice experience in the United States supervised the translation of the quotes used in this manuscript. Data collection continued until no new categories emerged (Mohammadi et al., 2020).

### 3.5 Data analysis

**FIGURE 1:**
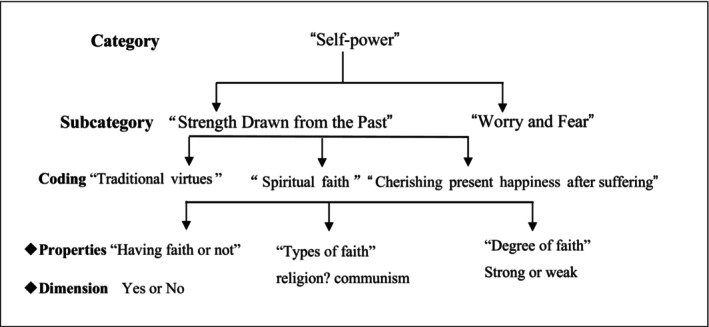
Seeking out properties and dimensions in open coding.

Analysis as an ongoing process was guided by the constant comparative method beginning with the first interview and continuing throughout the study. The first author read each transcript at least once to become familiar with the data. Transcripts were then analysed by a four‐stage constant comparison technique: open coding, axial coding, selective coding and theoretical integration using qualitative analysis software Nvivo Version 12. In open coding, the first author deconstructed data line by line to develop descriptive codes as labels for the meanings of the issues and ideas. The data were examined for concepts, categories and their properties and dimensions (Figure 1). In axial coding, categories were clustered together into meaningful, related groups by using the coding paradigm including ‘conditions, inter/actions and emotions and consequences’ to establish the connection between structure and process (Corbin & Strauss, 2015) (Table 2). During subsequent selective coding, all authors were involved in identifying the central construct and elements of subtle interactions until theoretical saturation. ‘A sense of responsibility’ related to empowering the older adults gradually emerged. ‘Responsibility endowing power’ became the core variable of health empowerment. Lastly, the themes are used to generate a theoretical framework to explain the data. ‘Memo‐writing’, or theoretical reflections was throughout the analysis process (Corbin & Strauss, 2008).

**TABLE 2:** An example of the coding paradigm.

Antecedent/Condition	Central phenomenon	Context	Intervention	Interaction	Consequence
Self‐power	Health responsibility initiation	Influencing factors: family/social power	Health responsibility performance: the five identified strategies	Self/family/society interaction	Health responsibility realization

### 3.6 Measures to enhance trustworthiness

Peer debriefing was completed via in‐depth discussion with PJ and ZH. They selected portions of transcripts to ensure the concurrence of coding. This was done regularly throughout the course of the study.

Credibility can be established through prolonged engagement with the interviewee. As stated earlier, the first author was immersed in the day care centre to establish a trustable interactive relationship with the older adults. Also, participants were invited to examine the findings and verify the emerging categories (Sikolia et al., 2013).

The analytic technique of waving the red flag was used for probing the data. When we were confronted with the seemed natural words such as ‘never’ or ‘always’ arise, which alerted us to investigate this claim further. For example, some participants stated they were always happy because they have a peaceful mind; researchers needed to explore what contributed to the result and whether there were other potential factors except for personality traits. Finally, five experts from nursing education, clinical medicine, community health and two older adults with chronic conditions (non‐interviewees) were invited to examine the categories and related items in the theoretical framework and modify the unsuitable items.

## 4 RESULTS

A total of 53 old participants with chronic conditions were investigated. The patients had a range of chronic conditions, including, cardiovascular disease (hypertension, coronary heart disease and arrhythmia), cerebrovascular disease, diabetes, chronic respiratory disease (chronic bronchitis, asthma and chronic obstructive pulmonary disease), chronic renal failure, cancer and osteoarthritis. Of these, 32 participants (58.5%) were affected by two to three coexisting health problems (Table 3).

**TABLE 3:** Participant background information.

	*N* = 53	%
City		
Shanghai	24	45.3
Nanjing	15	28.3
Bengbu	14	26.4
Age (years)		
60–69	20	37.7
70–79	16	30.2
80–89	16	30.2
>90	1	1.9
Sex		
Female	28	53
Male	25	47
Education		
Illiterate	6	11.3
Elementary	10	18.9
Junior high school	19	35.8
High school	10	18.9
Junior college	2	3.8
University	6	11.3
Marriage		
Widowed	14	26.4
Married (spouse paralysed)	36 (4)	67.9 (7.5)
Single/divorced	3	5.7
Income (RMB)		
<2000	13	24.5
2000–4000	25	47.2
4000–6000	6	11.3
6000–8000	2	3.8
8000–10,000	2	3.8
>10,000	5	9.4
Religion		
Buddhism	7	13.2
Christian	5	9.4
Taoism	4	7.5
None	37	69.8
Living with children		
Yes	28	52.8
No	25	47.2

**FIGURE 2:**
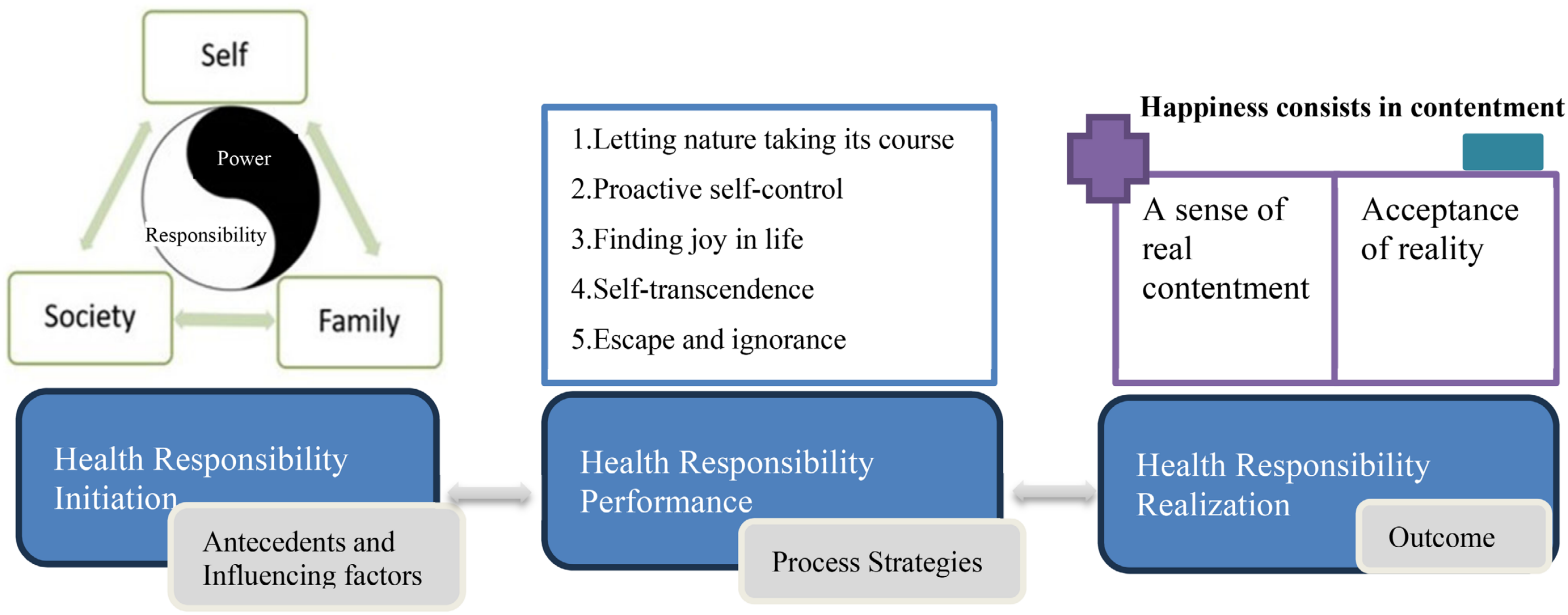
A theoretical framework of health empowerment for the Chinese older people with chronic conditions.

Totally, 205,000 words were transcribed and more than 700 codes were identified after the iterative process. Our findings indicated ‘responsibility endowing power’ emerged as the core theme overarching the following six themes: ‘health responsibility initiation’, ‘health responsibility performance’, ‘health responsibility realization’, ‘self’, ‘family’ and ‘society’. Of which, ‘self’, ‘family’ and ‘society’ contained dual ideas of power and responsibility. Table 4 showed all themes, categories and subcategories. The interaction between self, family and society constitutes the antecedents and influencing factors to initiate individual health responsibility, which contributes to the process strategies of performing the individual health responsibility, then predicting the consequence of realizing the individual health responsibility. In a word, health empowerment for older Chinese adults was a process of initiating, performing and realizing responsibility for health through the interaction between self, family and society (Figure 2).

**TABLE 4:** Supporting data of categories and subcategories.

Categories	Subcategories	Quotations
Theme 1: Health responsibility initiation: is defined as the interaction between the power of self, family and society, corresponding with the responsibilities of self, family and society, constituting the antecedent condition and influencing factors to initiate the health responsibility		
Theme 2: Self: means the dual power from and responsibility for self		
Self‐power	Worry and fear	B6: ‘It would be terrible if I couldn't take activities daily independently. Nobody can look after me. My children have their own children… I can only rely on myself and take good care of myself…’ B10: ‘I am a little pessimistic because my friends and neighbors were affected by strokes over the past years. The effects of stroke are too serious.… I can't imagine’. N1: ‘I am scared to give burden to my children if I fall since I always feel dizzy. …, children are very busy, and I don't want to trouble them’.
	Strength drawn from the past	S9: ‘I have taken care of myself for many years. I am happy now because I have rice to eat. You don't know the terrible natural disaster in the past, we were very hungry… Today I can buy anything I like’. B4: ‘I don't think I am ill. It does not matter. In the past, we had to eat grass rootsgrassroots. Nowadays we have enough food and clothes’. S7: ‘I learned much from my mother. She fostered me alone and told me to be a good man, I'll never forget my mother's love and instructions, it was my mother that gave me the greatest help in my life’. S6: ‘Since I am the oldest among children, I must be a good model for my younger sisters and brothers, which has developed my strong responsibility and self‐esteem’.
Self‐responsibility	Responsibility for self‐management of self‐help	N1: ‘when It is you who suffers illness, nobody else can help unless yourself because you know most yourself’. S6: ‘Maybe sometimes you can't help others sometimes, but you can help yourself as long as you decide to do’. B9: ‘I must keep well, to avoid getting others into trouble. I'd rather die than become a burden, one should live with dignity, … I always take care of myself, and do physical exercises to keep fit’. S17: ‘One must be responsible for self‐management. Hmm, I take care of myself well, which means I can help my family and government, I am happy’.
Theme 3: Family: means the dual power from and responsibility for family members		
Family power	From spouse From children and grandchildren From sibling	S12: ‘My husband is so kind. I could not stay well without his love. I always tell myself that I would do anything for him’. S15: ‘I am O.K. My son and daughter‐in‐law treat me well. I was content despite the fact that the illness could not be cured’. B5: ‘My children and grandchildren often visit and have a meal with me. I am always very happy to wait and cook for them when they arrive’. S21: ‘My grandson often brings me gifts and talks with me, he grows up and has a job, he always remembers to see me, what a good boy!’ S8: ‘My sister and brother often come to see and comfort me, I feel warm’.
Family responsibility	Responsibility for family members	S2: ‘I am holding on, just for my family. If I am alive, my family is a whole, once when I …, my family is incomplete…so I nearly live for my family’. S6: ‘…since I am the oldest and must plan for others. It is my responsibility to care for my family members…’ N5: ‘Good mood doesn't mean good family. But a good family can improve my good mood. They are interrelated. Family is important’. B6: ‘children's happiness is my happiness’ ‘I prefer to cook for my children, rather than they cook for me. This is my joy’. B12: ‘My granddaughter is my hope, like my Hope Project, As long as I can look after her, I am pleased. I hope to see her growing and her success in the future’.
Theme 4: Society: means the dual power from and responsibility for the society		
Social power	Accessible health resources	N10: ‘Perhaps you know yourself, but you are not familiar with the dose. You need to follow the doctor's orders, don't you?’ B8: ‘At present, I almost distrust that they (doctors and nurses) could do well. It is common to find their bad attitude ……The ordered drugs are useless; I prefer to believe in my own decision. If I were the leader of the ministry of health, I must condemn them for their medical immorality’. S14: ‘X hospital is very extraordinary; as you know they never respect our feelings. When we ask some questions, they are rude and interrupt us: ‘who is the doctor, you or I? ……Since you can treat your own illness, don't come here……’ We have no way’. S9: ‘We are not satisfied with the medical treatment, especially surgery; doctors always ask for a red packet (red envelope containing money as a bribe or gift) and extra fees, if you don't give them enough, they won't try their best to treat us’.
	Welfare policy	S9: ‘we must rely on the government, or else you can't live well’. S19: ‘Free riding, free parking tickets, and medical fees, the government gives us more consideration and frequently helps us. We should try to take care of ourselves and relieve the social burden’.
	Living environment	S17: ‘Many people don't respect older adults. Nowadays, if a grandma collapses on the street, nobody comes to help her.… This is common. China is an ancient civilized country; how could this be? This affects my health’. S5: ‘The old housing equipment and the quality of air influence my life, if these can be addressed by the government, we are perhaps better…’ B11: ‘I am unpleasant with the ward condition, with noise and crowd, the washroom is always dirty, why does the hospital not improve it?’
	Social contact	S2: ‘I gain a lot from the cancer club, particularly the teacher is also a patient with cancer, but he is compassionate to share knowledge… I am gradually changing. Now I am glad to share my experience and help others, my sister is surprised at my transition, Yes, I am happier when helping others’. S6: ‘I am lucky to enter the daycare center, we are not lonely, you are kind to accompany us and listen to us’.
Social responsibility	Responsibility for society Reward to society	S14: ‘Since the government gave me so much care, I hope to dedicate myself to society. If I can I will do so without any reward’. B12: ‘I am proud of China's prosperity and strong, I am glad to devote myself to my country if I can’. S5: ‘Serving people with a heart is the happiest of my life. If only sb needs my help, I'll come immediately’.
Theme 5: Health responsibility performance: are various behavior strategies used in performing health management by these participants.		
Letting nature take its course	B8: ‘Uh…go with the flow, we must follow the natural law’. B10: ‘letting nature take its course, I am aging’. B6: ‘Is there a better way to cure hypertension? No hospital in the world can cure it. Well, I adapt to it and never let it control me’.	
Proactive self‐control	S1: ‘I absolutely follow the doctor's orders. We must trust them’. B10: ‘Mind your own mouth and mind your own legs’.	
	B5: ‘I always care for my condition, once I feel sick, I went to see a doctor’. N2: ‘Every day I do exercise, I like to go hiking and practice TaiChi. After doing them, I feel relaxed and comfortable’.	
Finding joy in life	N12: ‘Anyway, I always like to find something to do when I am free. You must adjust yourself and find joy in life. I read books and newspapers; collect articles I like to enjoy the time’. B3: ‘A job is my spiritual console. Staying home is easy to fuss and uncomfortable. Once starting work; I am strong and forget all discomfort’. S20: ‘I am well when I go out to travel, once my diabetes was quiet serious, my husband and I traveled to Shaoxing, a good place to go to enjoy, we were very happy there’. B1: ‘You must adjust yourself and find joy in life. When I'm free, I read books and newspapers, cut out articles I like, I may not read in the future, but I enjoy the time’.	
Self‐transcendence	B9: ‘I had a bad temper in the past. Now I seldom lose my temper. It seems people with a bad temper are susceptible to diabetes. Since I believe in Buddhism, I always keep a good mood, which is better for my blood flow…’ S7: ‘I like Taoism; the book ‘dream of red mansions’ told us three principles: well, over, forget it, they are great, forget anything bad or useless… we should balance self. I also believe in Christianity, yesterday is a history, ah, tomorrow is a mystery. Today is a gift, also called a present! I think that whatever religion you believe in, you should be kind’. S3: ‘Remember to be benevolent to others, it's a Buddhism wisdom …’	
Escape and ignorance	B8, N3, S12: ‘No onset, no seeing a doctor’. B9: ‘…my lifestyle has last lasted in me for many years, why to change it? It is not good; people will die earlier (if changed)’. B13: ‘If my blood pressure is not high, I'll never notice’.	
Theme 6: Health responsibility realization: is the consequence of the older adults' empowerment after initiating and performing their health responsibility		
Happiness consists in contentment	A sense of real contentment	B10: ‘Diabetes or hypertension is not terrible, trying to control diet, look; all is not terrible, am I not well?’ S10: ‘Now, life is better than the past, you know, wages are as before and although we have retired, even more subsidies .ah, it's very good. Ha, I feel happy from the heart, very happy, I can stay with my husband and children. I feel healthy’. N11: ‘We are aging and have done all that should be done, try to live each day happy now, uh…happiness lies in contentment’.
	Acceptance of reality	B2: ‘There is no so‐called satisfaction or nonsatisfaction, I have accepted it, and just so…’ S13: ‘We have no excessive demands; clothing and food are enough, which is OK’. N15: ‘You must endure, or what can you do? Anyway… let it be’.

### 4.1 Health responsibility initiation

In this study, health responsibility initiation resulted from the interaction between the power of self, family and society, corresponding with responsibilities of self, family and society.

### 4.2 Self‐power/responsibility

*Self‐power* was referred to as a worry and fear for dependence and individuals' strength from past experience.

*Worry and fear*: Most participants acknowledged the fear and worry about losing self‐care and becoming a heavy family burden. ‘It would be terrible if I couldn't take activities daily independently. Nobody can look after me. My children have their own children… I can only rely on myself and take good care of myself…’ (Participant B6); ‘I am a little pessimistic because my friends and neighbors were affected by strokes over the past years. The effects of stroke are too serious.… I can't imagine’. (Participant B10). The worry and fear of becoming a burden facilitated self‐responsibility and independence.

*Strength drawn from the past*: Past experiences and suffering, such as past poverty, hunger and turmoil made the older adults cherish their present happiness. A participant with heart disease who was a soldier of the Liberation Army made the following statement: ‘I don't think I am ill. It does not matter. In the past, we had to eat grass roots. Nowadays we have enough food and clothes’. (Participant B4). A 93‐year‐old woman said, ‘I have taken care of myself for many years. I am happy now because I have rice to eat. You don't know the terrible natural disaster in the past, we were very hungry… Today I can buy anything I like’. (Participant S9).

Self‐responsibility: Most of the participants had the awareness of responsibility for self‐independence and self‐help. Traditional Chinese virtues, such as diligence and independence, foster a responsibility towards others and themselves. ‘One must be responsible for self‐management. Hmm, I take care of myself well, which means I can help my family and government, I am happy’. (Participant S17). ‘I must keep well, to avoid getting others into trouble. I'd rather die than become a burden, one should live with dignity…’ (Participant B9).

### 4.3 Family power/responsibility

Filial piety, spousal and sibling support were the major family power motivating health responsibility. On the other hand, participants wished to take some responsibility for the family while obtaining family support. ‘I am holding on, just for my family. If I am alive, my family is a whole, once when I …, my family is incomplete…so I nearly live for my family’. (S2).

Participants looked forward to staying with their children. ‘I am O.K. My son and daughter‐in‐law treat me well. I was content despite the fact that the illness could not be cured’. (Participant S15); ‘children's happiness is my happiness’ aptly described the feeling of many parents. They were proud of their children's success at work. Spousal affection was an important source of enhanced responsibility and contentment. One woman with ovarian cancer said affectionately, ‘My husband is so kind. I could not stay well without his love. I always tell myself that I would do anything for him’. (Participant S12). As they stated ‘Good mood doesn't mean good family. However, a good family can improve my good mood. They are interrelated’. (Participant N5). The harmonious relationship between family power and family responsibility directly influences the participants' well‐being outcome.

### 4.4 Social power/responsibility

*Social power* in this study referred to accessible health resources, welfare policies, living environment and social contact.

*Accessible health resources*: Doctor–client relationships or community services affected the access to health resources. Participants emphasized the importance of the doctor–client relationship and expected to cooperate with the health professionals and receive effective instructions. ‘Perhaps you know yourself, but you are not familiar with the dose. You need to follow the doctor's orders, don't you?’ (Participant N10).

The participants respected and appreciated the skilled and amiable professionals who were glad to communicate with the patients. Some participants whose children worked in a health care agency felt fortunate for the help received. For instance, a participant with kidney cancer whose children were medical professionals represents a typical case of a patient gaining prompt access to diagnosis and treatment. Many participants wished their children to be professionals in the future to easily see a doctor. Participants in more developed Shanghai were satisfied with the well‐equipped community service resources with health instructions, reading clubs, free physical check‐ups organized by neighbourhood committees and senior day care centres.

*Welfare policy*: The older adults attributed their health responsibility to national health insurance and special welfare policies for older adults. ‘Free riding, free parking tickets, and medical fees, the government gives us more consideration and frequently helps us. We should try to take care of ourselves and relieve the social burden’. (Participant S19). ‘We can't live well without our great government’. (Participant S9).

*Living environment*: The poor quality of the air and water, some old housing environment and social disrespect towards older adults were living environmental factors inhibiting older people from performing health responsibilities to achieve their happiness. ‘Many people don't respect older adults. Nowadays, if a grandma collapses on the street, nobody comes to help her.… This is common. China is an ancient civilized country; how could this be? This affects my health’. (Participant S17).

*Social contact*: The support from friends and peers was recognized as social contact which empowered participants. A participant described peer support as follows, ‘I gain a lot from the cancer club, particularly the teacher is also a patient with cancer, but he is compassionate to share knowledge… I am gradually changing. Now I am glad to share my experience and help others, my sister is surprised at my transition, Yes, I am happier when helping others’. (Participant S2).

*Social responsibility* meant a sense of responsibility for society and their willing of reward to society. For participants, national destiny was closely interrelated to their wellness. They were proud of the prosperous and rich motherland and wished to work for society. ‘As the government cared for me, I hope to dedicate myself to society. If I can I will do so without any reward’. (Participant S14). ‘Serving people with a heart is the happiest of my life. If only sb needs my help, I'll come immediately’. (Participant S5).

As stated above, the power of self, family and society motivated the formulation of health responsibility, which became the antecedent condition and influencing factors of health empowerment.

### 4.5 Health responsibility performance

Health responsibility performance was referred to as the process of accomplishing health management by older adults with chronic conditions. Five behaviour strategies were identified as followed.

*Letting nature take its course*: Participants seemingly adopted a ‘natural attitude’ or wisdom towards illness and aging by statements of ‘go with the flow, we must follow the natural law’. (Participant B8) or ‘letting nature take its course, I am aging’. (Participant B10). ‘Is there a better way to cure hypertension? No hospital in the world can cure it. Well, I adapt to it and never let it control me’. (Participant B6).

*Proactive self‐control*: Adopting a natural attitude did not mean submitting to fate, participants attempted to control diseases by positive self‐monitoring instead. They positively consulted the doctor and followed the advice of the staff. ‘I absolutely follow the doctor's orders. We must trust them’. (Participant S1). The proverb ‘Mind your own mouth and mind your own legs’ explained diet and exercise management. Traditional remedies such as massage, acupuncture, Tai Chi and regular check‐ups were prevalent. ‘Every day I do exercise, I like to go hiking and practice TaiChi. After doing them, I feel relaxed and comfortable’. (Participant N2).

*Finding joy in life*: Life was more than disease treatment. The participants were happy to find joy by travelling, gardening, looking after grandchildren and reminiscence to create a purposeful life. ‘Anyway, I always like to find something to do when I am free. You must adjust yourself and find joy in life. I read books and newspapers; collect articles I like to enjoy the time’. (Participant N12). A participant who obtained a job after retirement said, ‘Job is my spiritual console. Once starting work, I felt strong and forgot all discomfort’. (Participant B3).

*Self‐transcendence*: Self‐transcendence (ST) referred to a characteristic development characterized by the physical, psychological and social expansion of self‐boundaries, broadening one's life perspective and purposes, which is related to emotional well‐being (Wright, 2003). In our framework, qualities from religion and traditional cultural wisdom, including giving thanks, tolerance and kindness towards others were classified as forms of ST to purify the mind and balance the self. A participant with diabetes believed that Buddhism created a mood of tranquillity and intelligence, ‘I had a bad temper in the past. It seems that people with a bad temper are susceptible to diabetes. Since following Buddhism, I am always in a good mood, which is better for my blood flow…’ (Participant B9). A respondent with Christian faith also appreciated Taoism: ‘I like Taoism; the book ‘dream of red mansions’ told us three principles: well, over, forget it, they are great, forget anything bad or useless… we should balance self. I also believe in Christianity, yesterday is a history, ah, tomorrow is a mystery. Today is a gift, also called a present! I think that whatever religion you believe in, you should be kind’. (Participant S7).

*Escape and ignorance*: While some participants performed positive self‐management, others did not take any medication or undergo physical check‐ups unless their symptoms were too serious to control by themselves. ‘No onset, no seeing a doctor’ (Participant B13) or ‘If my blood pressure is not high, I'll never notice’.(Participant B8). They continued smoking, alcohol and greasy or pickled food consumption and recognized the harmful behaviour they were nurturing. ‘…my lifestyle has lasted in me for many years, why to change it? It is not good; people will die earlier (if changed)’. (Participant B9).

This kind of behaviour of ignoring responsibility for one's own health reflects an old Chinese proverb ‘never shed tears until seeing the coffin’. Perhaps it also reflected the inaccessibility to health resources to some degree.

### 4.6 Health responsibility realization

Health responsibility realization was referred to as the consequence of the older adults' empowerment after initiating and performing their health responsibility.

### 4.7 Happiness consists in contentment

In our study, participants had a comprehensive understanding of health empowerment. They most commonly mentioned the statement ‘happiness consists in contentment’ which was categorized as the ‘health responsibility realization’, ‘happiness consists in contentment’ with a dual meaning was regarded as the consequence of health empowerment, including a sense of real contentment or an acceptance of reality.

*A sense of real contentment* signified a positive attitude towards successfully accomplishing health responsibility. ‘Diabetes or hypertension is not terrible, trying to control diet, look; all is not terrible, am I not well?’ (Participant B10) ‘Life is better than the past, you know, wages are as before and although we have retired even more subsidies. Ha, I feel happy from the heart, I can stay with my husband and children. I feel healthy’. (Participant S10).

*Acceptance of reality*: ‘Happiness consists in contentment’ mentioned by older adults also implied that they never had extravagant hopes after experiencing earlier vicissitudes. There was neither being satisfactory nor being dissatisfactory. ‘We have no excessive demands; clothing and food are enough, which is OK’. (Participant S13). ‘You must endure, or what can you do? Anyway… let it be’ (Participant N15), which indicated more acceptance of reality than contentment.

## 5 DISCUSSION

This study described an empowerment framework for the Chinese older adults by identifying ‘responsibility endowing power’ as the core category, which highlighted the vitality of responsibility in empowerment. However, the responsibility did not arise independently. Our study indicated that it was the interaction between ‘self, family and society’ that initiate the health responsibility of the older people, and then responsibility motivated the power, constituting the antecedent and influencing factors of health empowerment. The empowerment process was ‘health responsibility performance’, involving five strategies as stated above. ‘Health responsibility realization’ as the consequence of the older adults' empowerment after initiating and performing their health responsibility, includes ‘happiness consists in contentment’ with dual meaning.

### 5.1 Initiating health responsibility with the interaction between self, family and society

This finding indicated a health responsibility stemmed from the interaction between self, family and society with a symbiosis of power and responsibility, which formulated the antecedents and influencing factors of health responsibility initiation. Conversely, the achievement of health responsibility influenced the family, self and society.

Empowerment varies with cultural differences (Yip, 2004). In our study, participants viewed family affection and patriotism as inseparable components of a healthy life, which was limited in other studies. Traditional Chinese Confucianism advocated family–country‐based self‐cultivation. The responsibility for the family is a part of an individual's spiritual belief (Zhou & Wu, 2010). A study by Crawford Shearer and Reed (2004) involving older women, described health as the ability to care for oneself and help others. The fear of being a burden on the family facilitated a sense of self‐help responsibility. An Iranian diabetic empowerment study obtained a similar result (Abdoli et al., 2008). Older people in the current study always cared for the fate of the nation. This shows empowerment for individual Chinese means both rights and responsibility within one's social roles. The Chinese elders' responsibility for society and family would be further strengthened by love from society and family in such a way as to strengthen the responsibility for self‐help to achieve empowerment, which explained ‘social self’ as a key part of the self. Once having responsibility, they have the power. The responsibility for self meant contributing towards the family and country and entailed love from society and the family to achieve empowerment, which revealed how to interpret self in a social context (Benzies & Allen, 2001).

External social factors acting on self were indispensable for empowerment. Deficiencies in the medical environment inhibited empowerment. Generally, involvement in decision‐making by consulting with health professionals was essential for empowerment (Stacey et al., 2017). However, participants in our study did not present a strong desire for decision‐making participation and remained dependent on the physician; Náfrádi et al. (2017) also indicated patients' self‐efficacy and internal health control joint with physician‐attributed control beliefs constituted an empowerment approach to promote medication adherence. Some participants hoped to participate in decision‐making but had no opportunity, which explained why participants wished that their kids were medical staff for health resources accessibility. Meyer et al. (2008) noted that gaining access to health care resources precedes expressing choices and decision‐making. Our study added to the evidence.

### 5.2 Health responsibility performance as process strategies of health empowerment

Participants adopted five positive or negative behaviours to sustain health responsibility. The seemingly negative ‘letting nature take its course’, combined with ‘proactive self‐control’ was comparable to Aujoulat et al.'s (2008) dual empowerment opinion of accepting anything uncontrolled. Empowerment may mean ignoring illness but attaching importance to health. ‘Finding joy in life’ full of creativity and ‘self‐transcendence’ in our study connoted a sense of ‘purpose of life’. Lundman et al. (2010) concluded that people with a high degree of purpose in life were free to choose their attitudes towards everything in all life moments and reach out beyond themselves with inner strength, which embodied the intrapersonal level of health empowerment. Significantly, studies (Náfrádi et al., 2018; Schulz & Nakamoto, 2013) have revealed that an empowered patient with high health literacy is an effective self‐manager, while an empowered patient who is not a health literate might be a dangerous self‐manager. Dangerous self‐managers are inclined to engage in non‐beneficial health behaviours potentially leading to low health outcomes. Our findings supported this statement and verified that some people with ‘self‐escape and ignorance’ had a concurrent performing ‘finding joy in life’ to distract from symptoms.

### 5.3 Health responsibility realization as a consequence of health empowerment

‘Happiness consists in contentment’ was defined as the realization of health responsibility by the participants. ‘Happiness consists in contentment’ derives from Chinese Taoism, a dialectic philosophy that upholds the natural and dynamic balance. When the self, family and society acted together perfectly, ‘happiness consists in contentment’ meant the real realization of health responsibility with power. For another, it was possibly linked with endurance or having to accept the second best, with relinquishing the sense of control. This seemingly coincided with self‐transcendence theory advocating personal‐boundaries expansion towards maturation and wisdom by redefinition of self rather than an overemphasis on the disease (Wright, 2003). Health empowerment for Chinese older people should ensure older people have access to sources to achieve contentment and realize their health responsibility. Of interest, health providers should distinguish the beneficial or non‐beneficial behaviour when providing empowerment intervention.

### 5.4 Implications

The results of this study shed light on the definition, antecedents, process, and outcome of health empowerment for Chinese older adults with chronic conditions, with an implication of developing effective nursing strategies to initiate the inner power and confidence of the Chinese older people for self‐help and self‐management. Based on our theoretical framework, family care and social support were believed to be indispensable variables and should be involved in the health empowerment program. Health care providers should advocate the comprehensive strengthening of community service and a social and humanistic environment for older adults' health empowerment. In consideration of the experience of interaction with older people by the research, further narrative or story‐telling intervention in addition to self‐management skills should be involved in the further empowerment intervention to promote self, family or medical interaction. To measure the efficacy of health empowerment for older people, a specific measurement scale should be designed based on the framework.

### 5.5 Limitations

This theoretical framework of health empowerment was rooted in Chinese culture with specific space–time characteristics. With the social and cultural development, the health perspectives of older adults may vary with generations. Considering the limitation of a qualitative study, the generalization of the findings should be cautious. Continuous revision of the framework is needed as time passes.

## 6 CONCLUSION

The study contributing to the theoretical understanding of ‘responsibility endowing power’ enriches the meaning of health empowerment by expounding on the significance of responsibility in health empowerment. Nurses should help individuals be aware of health responsibility from the following three aspects, namely self, family and society. Similarly, to achieve the outcome of contentment, older people and their family may be resourceful in seeking healthy interaction and accessible supportive resources through effective empowerment strategies. Further work should develop an empowerment program for the Chinese older people and measurement instruments based on the theoretical framework.

## FUNDING INFORMATION

The author(s) disclosed receipt of the following financial support for the research, authorship and/or publication of this article: This work was supported by Universities' Philosophy and Social Science research project in Jiangsu Province, China (grant number 2021SJA0335).

## CONFLICT OF INTEREST STATEMENT

No conflict of interest has been declared by the author(s).

## Data Availability

The data that support the findings of this study are available from the corresponding author, Zhang H, upon reasonable request.
